# Rewriting the RNA code: an m^6^a-centric framework to classify tumors and guide combination therapies

**DOI:** 10.3389/fimmu.2026.1749911

**Published:** 2026-01-30

**Authors:** Yi Sun, Jinliang Wu, Guanhao Chen, Haojun Ma, Wenshuya Li, Hongyu Tan, Kerong Yang

**Affiliations:** 1 Department of Breast Surgery, The First Affiliated Hospital of Zhengzhou University, Zhengzhou, Henan, China; 2 Department of Orthopedics, The First Affiliated Hospital of Zhengzhou University, Zhengzhou, Henan, China; 3 The First Clinical School of Medicine, Zhengzhou University, Zhengzhou, Henan, China; 4 Department of the Plastic Surgery, The Second Hospital of Hebei Medical University, Shijiazhuang, China

**Keywords:** cancer immunotherapy, epitranscriptome, N^6^-methylAdenosine (m^6^A), RNA modification, targeted therapy, therapeutic resistance

## Abstract

**Background:**

The epitranscriptome, particularly N^6^-methyladenosine (m^6^A), represents a dynamic layer of post-transcriptional regulation fundamentally implicated in cancer. However, the clinical translation of this knowledge is hampered by profound context-dependency, where the same m^6^A regulator can exert opposing roles in different tumors. To overcome this barrier, we propose a novel, clinically actionable taxonomic framework that classifies tumors based on their dominant dysregulated m^6^A component.

**Methods:**

We synthesized current evidence through systematic reviews of primary research and high-impact papers from PubMed and Google Scholar, focusing on the mechanistic role of m^6^A modifications in cancer biology, therapy resistance, and therapeutic targeting. This synthesis was used to integrate pan-cancer molecular data including regulator expression, genetic dependency scores, and modification landscapes to define and characterize m^6^A-driven molecular subtypes.

**Results:**

We classify tumors into Writer-Dominant (METTL3/14-high, Eraser-High (FTO/ALKBH5-high), Reader-Amplified (IGF2BP/YTHDF-high), and Immune-Modulatory subtypes, each with distinct oncogenic programs, therapy resistance mechanisms, and, crucially, actionable therapeutic vulnerabilities. We provide explicit, evidence-based molecular and functional inclusion criteria for each subtype and acknowledge that tumors can exhibit hybrid features, which directly inform rational combination strategies. Furthermore, we detail a diagnostic-therapeutic roadmap that integrates liquid biopsy-based m^6^A biomarker detection with subtype-specific treatment assignment.

**Conclusion:**

Targeting the m^6^A epitranscriptome represents a paradigm shift in oncology; our framework provides the essential strategic approach needed to overcome context-dependency, offering a logical structure for tumor classification, vulnerability prediction, and the translation of epitranscriptomic insights into patient benefit through personalized, biomarker-guided combination therapies.

## 1 Introduction

For decades, the existence of chemically modified nucleosides in RNA has been known, but a deep appreciation of their extensive and sophisticated regulatory roles in cellular processes has only emerged in recent years (1). It is now clear that these post-transcriptional modifications impart a layer of functional diversity to the four standard ribonucleotides, analogous to the side chains of amino acids in proteins, thereby empowering RNA to form intricate structures and engage in specific molecular interactions. This extensive collection of chemical alterations, collectively termed the epitranscriptome, can directly influence RNA architecture-enhancing rigidity or flexibility-and critically modulate its interactions with proteins, thereby profoundly shaping complex regulatory networks and cellular metabolism (2).

Spurred by revolutionary technological advancements, especially in liquid chromatography/mass spectrometry (LC/MS)-based methodologies, the field has seen the discovery of novel RNA modifications and the definitive elucidation of the physiological functions of many known nucleosides (3–6). A truly pivotal discovery was the identification of reversible RNA methylation, which established the epitranscriptome as a highly dynamic regulatory system, parallel to the epigenome. A prime illustration is N^6^-methyladenosine (m^6^A), which plays a non-redundant and critical role in gene expression regulation by influencing transcript stability, splicing, translation efficiency, and cap-independent translation (7). The fundamental biological importance of this machinery is further highlighted by the direct association between mutations in human genes encoding RNA-modifying enzymes and a wide spectrum of diseases, including cancer, cardiovascular disorders, and neurological conditions (8).

This review will first synthesize the current understanding of the coordinated “writer-eraser-reader” machinery and detail the mechanisms through which its dysregulation propels oncogenic processes and therapy resistance. However, a critical barrier remains: the context-dependent functions of m^6^A regulators often lead to contradictory findings, creating a significant obstacle for developing unified therapeutic strategies. To bridge this gap between mechanistic insight and clinical application, the central thrust of this review is to introduce and elaborate a novel m^6^A-based taxonomic framework. This framework is grounded in the distinct, non-redundant functions of each component class (writers, erasers, readers) and their collective impact on the tumor immune microenvironment. It moves beyond a gene-centric view to define tumors by their dominant dysregulated m^6^A component, classifying them into clinically actionable subtypes with explicit molecular criteria. We will demonstrate how this taxonomy-comprising Writer-Dominant, Eraser-High, Reader-Amplified, and Immune-Modulatory subtypes provides a logical structure for understanding oncogenic mechanisms, predicting therapeutic vulnerabilities, and guiding rational drug combinations. Importantly, we will address the potential for tumors to exhibit features of multiple subtypes, providing a nuanced approach to classification and combination therapy. Finally, we will detail a diagnostic-therapeutic roadmap that integrates liquid biopsy-based biomarker development with this subtype-driven approach to therapy. By proposing and validating this integrative framework, this review aims to provide a coherent strategy for leveraging the epitranscriptome to achieve personalized cancer medicine.

## 2 Literature search strategy

To ensure a comprehensive and current overview of the field, the literature for this review was identified through a systematic search of the PubMed and Google Scholar databases, covering the period up to October 2025. The search strategy employed a combination of the following key terms and phrases: “N^6^-methyladenosine” OR “m^6^A” OR “epitranscriptome” in conjunction with “cancer” OR “oncogenesis” OR “tumorigenesis” OR “therapeutic resistance” OR “chemotherapy” OR “immunotherapy” OR “targeted therapy” OR “METTL3” OR “FTO” OR “ALKBH5” OR “YTHDF” OR “IGF2BP”.

The initial search results were screened by title and abstract for relevance. Primary research articles and high-impact reviews that elucidated the mechanistic role of m^6^A modifications in cancer biology, therapy resistance, and therapeutic targeting were selected for full-text review. The reference lists of key publications were also examined to identify additional pertinent studies. This strategy was designed to synthesize a timely and authoritative perspective on the dynamic role of the m^6^A epitranscriptome in cancer.

## 3 The m^6^A epitranscriptomic machinery: writers, erasers, and readers

**Figure 1:**
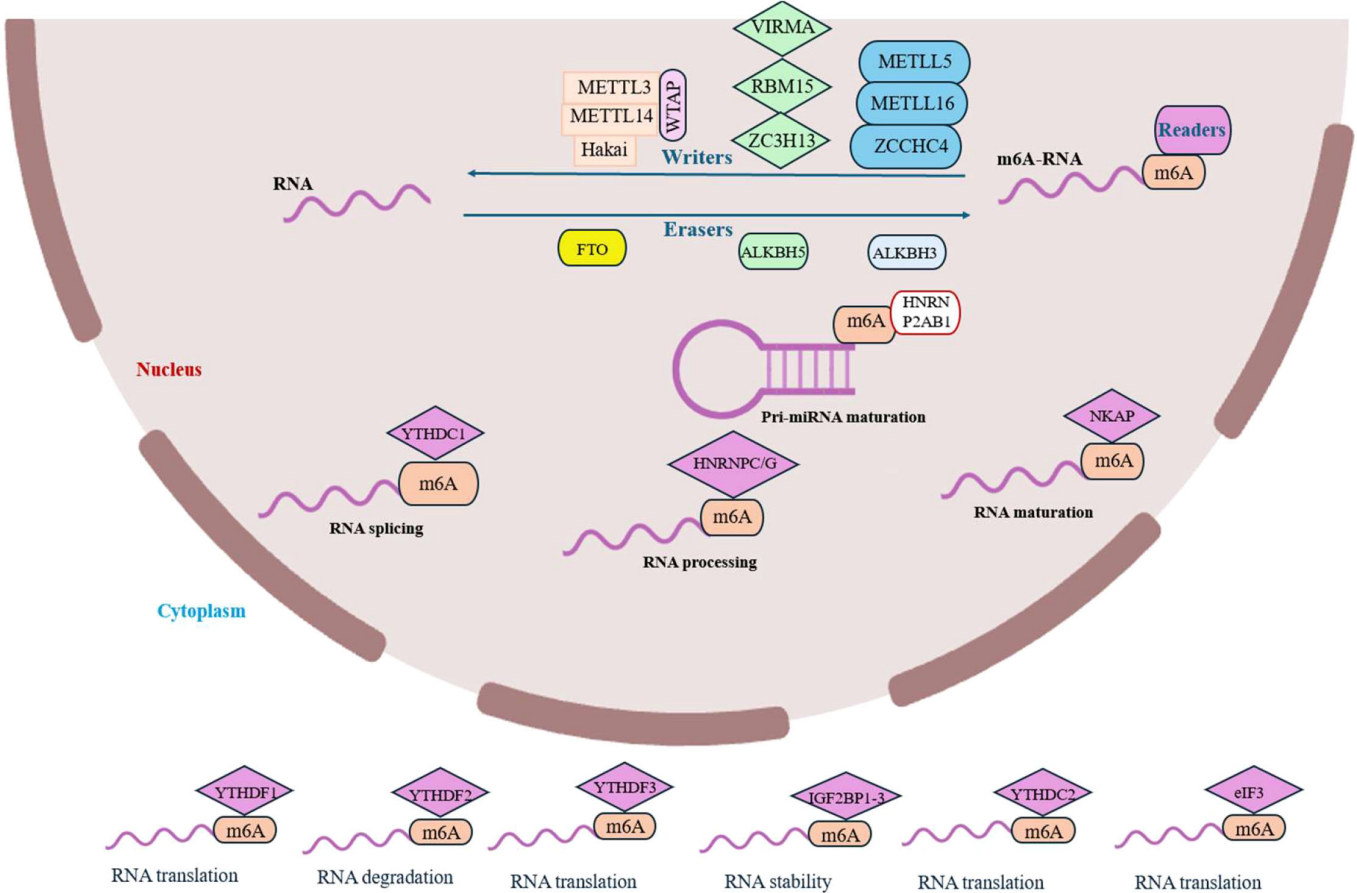
The m^6^A epitranscriptomic machinery: writers, erasers, and readers. The N6-methyladenosine (m^6^A) modification is dynamically regulated by a conserved set of proteins. The methylation (“writing”) is catalyzed by a multi-component methyltransferase complex, whose catalytic core is the METTL3-METTL14 heterodimer. This complex is guided and regulated by auxiliary proteins including WTAP, VIRMA, RBM15/15B, ZC3H13, and others, which determine its substrate specificity, localization, and stability. Demethylation (“erasure”) is performed by the Fe (II)/α-ketoglutarate-dependent dioxygenases FTO and ALKBH5, which oxidatively remove the methyl mark to reverse its function. The biological consequences of m^6^A are executed by “reader” proteins that specifically recognize the modified adenosine. These readers, which include members of the YTH domain family (YTHDF1-3, YTHDC1-2), IGF2BPs, and heterogeneous nuclear ribonucleoproteins (HNRNPs), dictate the fate of the target RNA by regulating its splicing, stability, translation, and subcellular localization.

N^6^-methyladenosine (m^6^A) is the most prevalent internal chemical modification in eukaryotic (messenger RNA) mRNA and long non-coding RNA (lncRNA), found across a diverse range of species from viruses and bacteria to plants and mammals (4, 5, 9–11). This dynamic and reversible modification is intricately involved in nearly every aspect of RNA metabolism, including pre-mRNA processing, nuclear export, translation efficiency, transcript stability, and miRNA biogenesis, thereby regulating critical biological processes such as development, the cell cycle, and stress responses (7). The installation, interpretation, and removal of m^6^A are governed by three specialized classes of proteins: ‘writers’ (methyltransferases), ‘erasers’ (demethylases), and ‘readers’ (m^6^A-binding proteins), which work in concert to fine-tune gene expression in response to cellular needs (**Figure 1**) (12–14).

The core writer machinery in humans is a multi-component methyltransferase complex. Its catalytic engine is the stable heterodimer formed by METTL3 and METTL14, both class I methyltransferases (15–17). This heterodimeric complex was identified as the central functional unit, where METTL3 and METTL14 form a stable 1:1 complex that is essential for cellular m^6^A deposition (18). While METTL14 alone possesses methyltransferase activity, its combination with METTL3 results in a dramatic, synergistic enhancement of catalytic function, underscoring their partnership as the core catalytic engine. Furthermore, the regulatory subunit Wilms’ tumor 1-associating protein (WTAP), which itself lacks catalytic activity, is a vital component that interacts with the METTL3-METTL14 heterodimer. WTAP is crucial for recruiting the complex to nuclear speckles and, critically, it significantly enhances the m^6^A methylation activity of the complex within cells (19–21). Structural studies reveal that the METTL3-METTL14 complex forms an asymmetric heterodimer where METTL3 contains the functional active site for S-adenosylmethionine (SAM)-dependent methyl transfer, while METTL14 serves primarily as a crucial structural scaffold that enhances METTL3’s catalytic activity and binds RNA (22). he complex is further regulated by several auxiliary subunits: RNA-binding motif protein 15 (RBM15) binds to METTL3 and WTAP and promotes mRNA nucleation; VIRMA (also known as KIAA1429) recruits the core complex to preferentially catalyze methylation in 3’UTRs and near stop codons (23); Hakai (CBLL1) is an E3 ubiquitin ligase that helps maintain complex stability (24); and ZC3H13 acts as a scaffold, connecting RBM15, WTAP, and VIRMA and regulating their nuclear localization (25, 26). Beyond this core complex, other methyltransferases perform specific functions: METTL16 regulates SAM homeostasis by methylating MAT2A mRNA and U^6^ small nuclear RNA (snRNA) and is implicated in DNA damage repair (27–29), while METTL5 and ZCCHC4 are responsible for methylating 18S and 28S rRNA, respectively (30, 31).

The dynamic nature of the m^6^A modification is enabled by eraser enzymes that oxidatively remove the methyl group. The two primary m^6^A demethylases, FTO and ALKBH5, are Fe (II)- and α-ketoglutarate-dependent dioxygenases from the ALKB family (32). Both enzymes possess shallow substrate-binding grooves that favor binding to single-stranded RNA (33). Cellular studies confirm their physiological role: FTO localizes to nuclear speckles and its knockdown increases cellular m^6^A levels, while its overexpression decreases them. Its activity is influenced by RNA sequence and tertiary structure and is chaperoned by the RNA-binding protein SFPQ, which recruits FTO to specific CUGUG sequences for proximal demethylation (34, 35). Similarly, ALKBH5 catalyzes m^6^A demethylation, and its knockdown also increases m^6^A levels. It colocalizes with nuclear speckles and is crucial for proper mRNA export and metabolism, with its biological significance highlighted by the fact that Alkbh5 deficiency in mice causes male infertility due to defective spermatogenesis. Another ALKB family member, ALKBH3, also exhibits m^6^A demethylation activity (36).

The functional consequences of m^6^A methylation are executed by reader proteins that specifically recognize the mark. The most well-characterized readers are the YTH domain family proteins. Cytosolic YTHDF1 promotes translation, YTHDF2 accelerates mRNA decay, and YTHDF3 plays a cooperative role with both-a topic of ongoing investigation regarding the precise redundancy versus specialization of these proteins (37, 38). Nuclear readers YTHDC1 and YTHDC2 regulate mRNA splicing, export, and translation. Beyond the YTH family, other proteins serve as readers. The Heterogeneous Nuclear Ribonucleoprotein (HNRNP) family includes HNRNPA2B1, which binds m^6^A-modified pri-miRNAs to recruit DGCR8 and promote miRNA processing (39). HNRNPC and HNRNPG function through an “m^6^A switch” mechanism, where m^6^A modification alters RNA structure to modulate their binding, thereby influencing RNA processing and metabolism (40). Recent evidence also implicates HNRNPR in m^6^A-mediated tumor glycolysis. The Insulin-like growth factor 2 mRNA-binding proteins (IGF2BPs; including IGF2BP1/2/3) recognize m^6^A marks to enhance mRNA stability and translation, often by recruiting co-factors like HuR (41). Additional readers include NF-κB activating protein (NKAP), which promotes mRNA splicing and maturation (42), and eukaryotic initiation factor 3 (eIF3), which binds m^6^A in the 5’UTR to facilitate cap-independent translation initiation (43). This intricate and diverse network of readers ensures that the m^6^A modification is translated into precise functional outcomes, regulating RNA fate at multiple levels.

## 4 Mechanisms of epitranscriptome-driven oncogenesis

### 4.1 Regulation of key oncogenes and tumor suppressors

**Figure 2:**
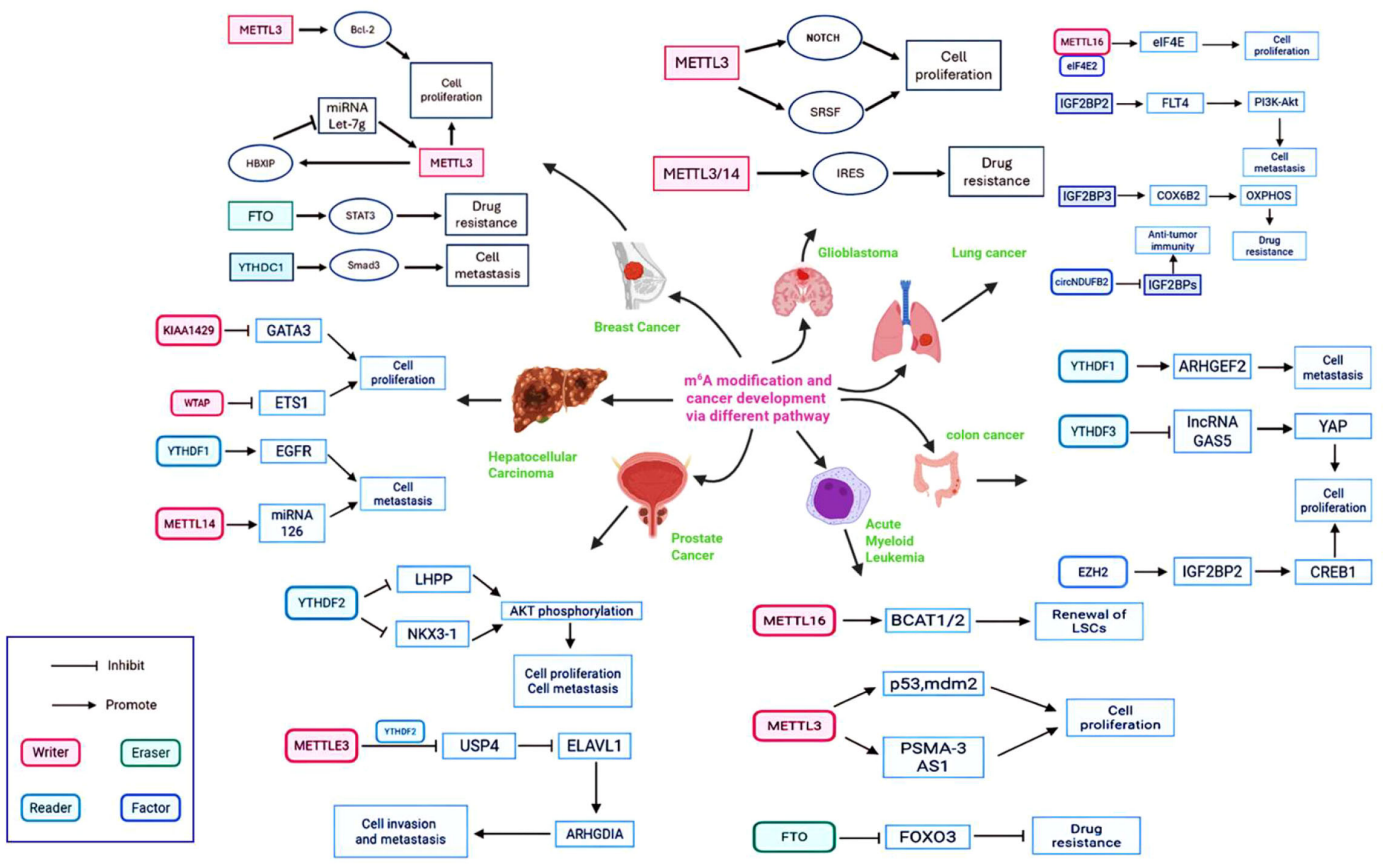
The oncogenic m^6^A regulatory network across cancer types. This figure summarizes the complex interactions between the core m^6^A machinery (Writers: METTL3, METTL14, METTL16, WTAP, KIAA1429; Eraser: FTO; Readers: YTHDF1/2/3, YTHDC1) and their key downstream targets. Arrows denote promoting effects, while blunt lines denote inhibitory effects. The network highlights how epitranscriptomic dysregulation drives fundamental cancer hallmarks-including uncontrolled proliferation, metastasis, and therapy resistance-in a variety of cancers (Acute Myeloid Leukemia, Breast Cancer, Glioblastoma).

The epitranscriptome exerts precise control over the life cycle of mRNAs encoding critical oncoproteins and tumor suppressors, thereby functioning as a master switch for malignant transformation. This control is not isolated but is part of a vast, interconnected regulatory network where writers, erasers, and readers coordinate to dictate tumor cell fate. As summarized in **Figure 2**, this intricate network demonstrates how the m^6^A machinery regulates a diverse array of oncogenic pathways-from the enhancement of MYC and BCL2 expression to the silencing of p53 and the activation of pro-metastatic signals like Smad3 and ARHGEF2-across a spectrum of cancer types. A prime example is the regulation of the MYC oncogene. In Acute Myeloid Leukemia the m^6^A writer METTL3 is recruited to the promoters of specific genes, leading to m^6^A methylation within the coding regions of their transcripts (44). This modification is critical for the efficient translation of key transcripts. For instance, METTL3-mediated m^6^A methylation of SP1 and SP2 mRNA does not affect their abundance but is essential for their translation (45, 46). The subsequent SP1/SP2 proteins then drive the expression of the MYC oncogene, creating a powerful feed-forward loop that sustains leukemogenesis (44). This simultaneous epitranscriptome-driven upregulation of oncogenes and downregulation of tumor suppressors creates a powerful driver of malignant transformation.

Beyond METTL3, the m^6^A writer METTL16 also plays a critical role in regulating MYC. In Hepatocellular Carcinoma (HCC), METTL16 is frequently upregulated and promotes tumor progression. It achieves this by installing m^6^A modifications on the long non-coding RNA RAB11B-AS1, which leads to the transcript’s destabilization and downregulation. The loss of this lncRNA creates a permissive environment for the expression of MYC, thereby fueling HCC growth and proliferation (47). This establishes METTL16 as a key upstream regulator of MYC in liver cancer. Conversely, the m^6^A eraser FTO plays an oncogenic role in specific AML subtypes by demethylating and destabilizing mRNAs of tumor suppressors like ASB2 and RARA (45). A summary of key m^6^A-regulated oncogenes and tumor suppressors across various cancer types is provided in **Tables 1** and **2**. This simultaneous epitranscriptome-driven upregulation of oncogenes and downregulation of tumor suppressors creates a powerful driver of malignant transformation.

**Table 1:** Pro-tumorigenic roles of m^6^A regulators via oncogene activation.

Cancer type	m^6^A regulator	Target gene	Mechanism	Functional outcome	References
Acute Myeloid Leukemia	METTL3	MYC	Enhanced translation via SP1/2	Promotes leukemogenesis	(44–46)
AML	FTO	MYC, CEBPA	Demethylation and stabilization	Promotes leukemogenesis	(45, 48)
Glioblastoma (GBM)	METTL3	ADAR1	Enhanced mRNA stability	Promotes growth & invasion	(49)
Hepatocellular Carcinoma (HCC)	METTL3	SOCS2	YTHDF2-mediated mRNA decay	Promotes growth & metastasis	(50)
HCC	METTL14	YTHDF2	Enhanced mRNA stability	Promotes metastasis	(50)
Gastric Cancer (GC)	METTL3	HDGF	Enhanced translation	Promotes growth & metastasis	(51)
GC	METTL3	MYC, EGFR	IGF2BP-mediated stabilization	Promotes proliferation	(52)
Colorectal Cancer (CRC)	METTL3	SOX2	Enhanced mRNA stability	Promotes stemness & chemoresistance	(53)
Breast Cancer	METTL3	BCL2, PTEN	YTHDF1-mediated translation/decay	Promotes growth & survival	(54)
Lung Cancer	METTL3	YAP	IGF2BP3-mediated stabilization	Promotes growth & metastasis	(55, 56)
Various Cancers	IGF2BP1/2/3	MYC, LIN28B	Recognition & stabilization of m^6^A-marked transcripts	Promotes proliferation, metastasis	(13, 41, 57)

**Table 2:** Pro-tumorigenic roles of m^6^A regulators via tumor suppressor silencing.

Cancer type	m^6^A regulator	Target gene	Mechanism	Functional outcome	References
Acute Myeloid Leukemia	FTO	ASB2, RARA	Demethylation and destabilization	Downregulation promotes leukemogenesis	(45)
Hepatocellular Carcinoma (HCC)	METTL14	LATS1, LATS2	YTHDF2-mediated mRNA decay	Promotes growth & metastasis	(58)
HCC	FTO	LATS1, LATS2	Demethylation and stabilization	Inhibits growth & metastasis (FTO acts as tumor suppressor here)	(48, 59)
Gastric Cancer (GC)	METTL3	PTEN	YTHDF2-mediated mRNA decay	Promotes proliferation	(60, 61)
Bladder Cancer	METTL14	NOTCH1	YTHDF1-mediated translation suppression	Inhibits progression (METTL14 acts as tumor suppressor here)	(62)
Glioblastoma (GBM)	ALKBH5	FOXM1	Demethylation and stabilization	Promotes growth & radioresistance	(63)
Breast Cancer	METTL3	BAX, P53	Altered splicing/translation	Promotes survival & chemoresistance	(64)
Endometrial Cancer	METTL14	PHLPP2	Enhanced mRNA stability	Inhibits AKT activation & tumor growth (METTL14 as tumor suppressor)	(65, 66)
Colorectal Cancer (CRC)	YTHDF1	TRIM29	Enhanced translation	Inhibits Wnt/β-catenin signaling (YTHDF1 as tumor suppressor)	(67, 68)

Conversely, the m^6^A machinery can also function to silence tumor suppressor genes, either through the erasure of stabilizing m^6^A marks or the writing of marks that promote the decay of tumor suppressor transcripts, thereby removing critical barriers to tumor progression.

### 4.2 m^6^A-mediated reprogramming of cancer cell metabolism

Cancer cells rewire their metabolism to support rapid growth, a process masterfully regulated by m^6^A RNA modifications. This epitranscriptomic reprogramming directly targets the core machinery of metabolic pathways, enhancing the stability or translation of key transcripts to fuel the Warburg effect and provide essential biosynthetic building blocks (27).

The Warburg effect describes the phenomenon where cancer cells preferentially utilize glycolysis for energy production, even in the presence of oxygen (69). Glycolysis decomposes glucose into pyruvate, a process involving numerous enzymes including hexokinase (HK), phosphofructokinase-1 (PFK), and pyruvate kinase (PK) (70, 71). While yielding less ATP than oxidative phosphorylation (OXPHOS) per glucose molecule, glycolysis operates at a much faster rate and generates intermediate metabolites crucial for synthesizing lipids, proteins, and nucleic acids, thereby supporting rapid proliferation (72). A key feature of this metabolic shift is the conversion of pyruvate to lactate by lactate dehydrogenase (73), which acidifies the tumor microenvironment and promotes immune escape by inhibiting the function of T and NK cells (74).

m^6^A methylation serves as a central regulator of this oncogenic metabolic switch. Transcripts encoding critical glycolytic enzymes, such as those for HK, PFK, and LDHA, are frequently modified by m^6^A, which enhances their translation efficiency and stability. For instance, the m^6^A reader YTHDF1 can bind to and promote the translation of glycolytic enzyme mRNAs, while IGF2BPs stabilize these transcripts to amplify their expression. This coordinated action significantly increases the flux through the glycolytic pathway. Furthermore, m^6^A modifications regulate transcripts involved in glutaminolysis and lipid synthesis, ensuring a steady supply of nitrogen and lipid precursors for membrane biogenesis. This epitranscriptomic control allows cancer cells to meet the heightened energetic and anabolic demands of uncontrolled proliferation, sustains a tumor-promoting microenvironment, and contributes to the clinical phenotypes observed in metabolic imaging (**Supplementary Table S1**) (24, 26).

### 4.3 Sustaining proliferative signaling and evading growth suppressors

The m^6^A machinery is integral to sustaining proliferative signaling, a dependency that can define specific molecular subtypes. A prime example is found in Acute Myeloid Leukemia, which often represents a prototypical “Writer-Dominant” subtype. CRISPR-Cas9 screens reveal that METTL3 is essential for the survival and proliferation of AML cells, while being dispensable for non-transformed cell. Its loss reverses the characteristic differentiation block of AML, promoting myeloid differentiation and apoptosis (75). This dependency is driven by METTL3’s catalytic activity, as a catalytically dead mutant cannot rescue the proliferation defect caused by its knockdown. METTL3 achieves this by regulating transcripts crucial for cell cycle progression and survival, such as BCL2 and MYC. Furthermore, METTL3 depletion in AML cells activates the PI3K/AKT pathway, as evidenced by increased phosphorylated AKT, which can contribute to differentiation signals (76). This establishes METTL3-high AML as a distinct entity with a unique therapeutic vulnerability to writer inhibition.

### 4.4 Promoting metastasis and invasion

The epitranscriptome is a key driver of cancer metastasis, particularly in solid tumors that can be classified as pro-invasive “Reader-Amplified” or “Eraser-High” subtypes. m^6^A modifications regulate the expression of transcripts critical for the epithelial-mesenchymal transition (77), such as the transcription factors SNAIL and ZEB1. By enhancing the stability or translation of these EMT master regulators, the m^6^A machinery promotes loss of cell adhesion and acquisition of migratory capabilities. Additionally, mRNAs encoding matrix metalloproteinases (MMPs) and integrins, which are essential for degrading the extracellular matrix and facilitating invasion, are common targets of m^6^A-mediated regulation, enabling cancer cells to invade surrounding tissues and vasculature (51, 52, 78). This epitranscriptomic reprogramming of the invasion machinery provides a functional signature for defining aggressive tumor subtypes.

### 4.5 Shaping the tumor immune microenvironment

Beyond cell-intrinsic effects, the epitranscriptome plays a crucial role in shaping the tumor immune microenvironment to enable immune evasion. The provided text highlights that FTO expression is upregulated by certain oncogenic proteins (MLL-fusion proteins, PML-RARA) in AML (45). While the direct immune checkpoint mechanisms are more prominent in solid tumors, the principle remains: m^6^A modifications precisely regulate the expression of immunomodulatory transcripts. For instance, the stability and translation of PD-L1 mRNA in tumor cells and PD-1 mRNA in T cells can be modulated by m^6^A, directly influencing the intensity of the immune response. Furthermore, cytokine signaling between tumor and immune cells is tuned by epitranscriptomic modifications, altering the inflammatory landscape of the tumor. This regulation also extends to innate immunity, influencing macrophage polarization and function within the tumor microenvironment (TME). This strategic manipulation allows cancer cells to create an immunosuppressive niche (79–81).

## 5 The role of the epitranscriptome in therapy resistance

**Figure 3:**
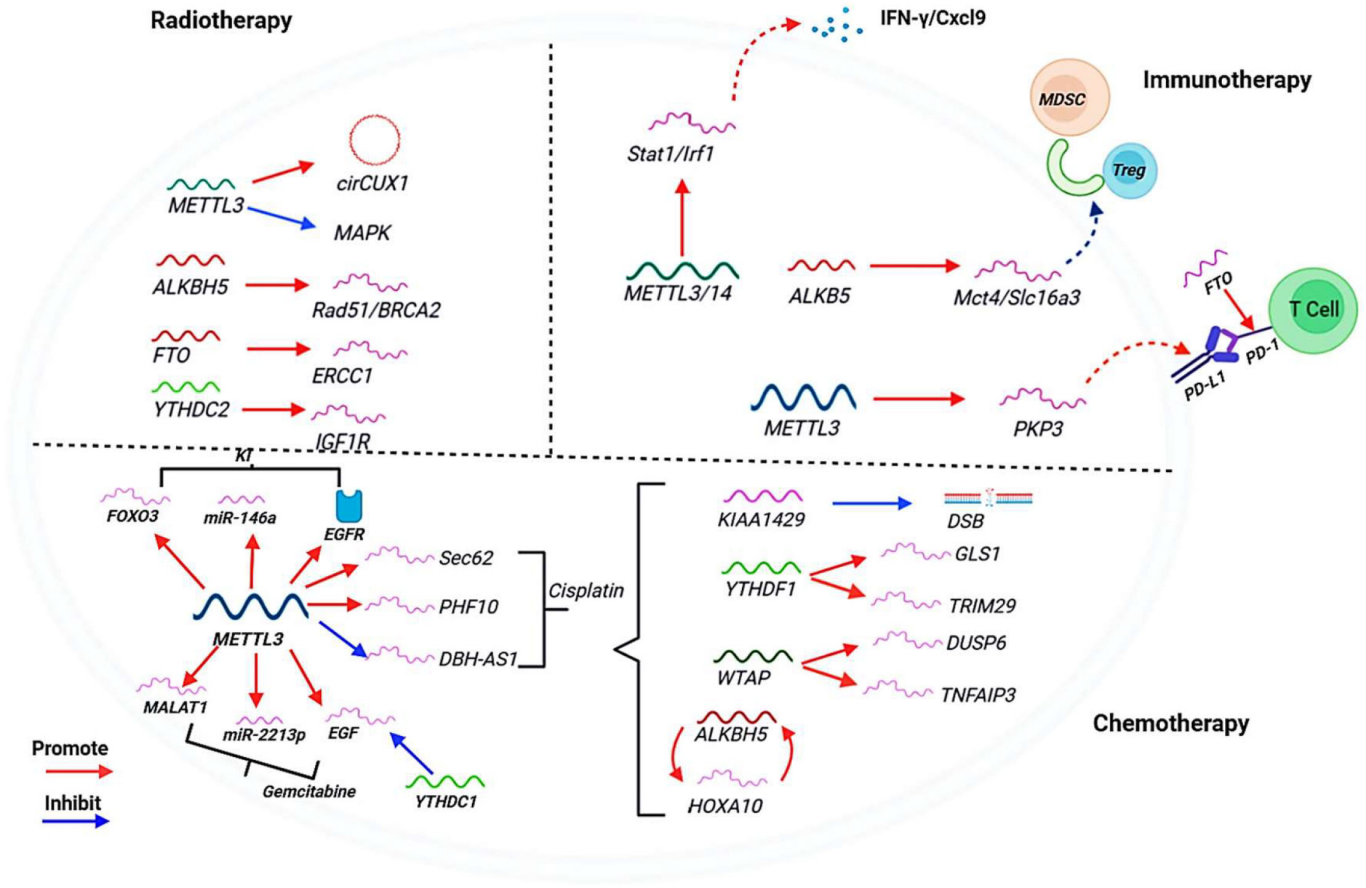
The role of m^6^A methylation in therapy resistance. The m^6^A regulators promote tumor cell resistance to radiation via stabilizing mRNA, regulating homologous recombination (HR) and inhibiting signaling pathways. The m^6^A regulators promote the stability of target gene mRNA and affect the recruitment of immune cells and the secretion of cytokines. The figure also outlines various pathways of m^6^A regulators in resistance to chemical drugs (Cisplatin, Gemcitabine, Kinase inhibitor). The red arrows indicate “promote” and the blue arrows indicate “inhibit”.

The epitranscriptome is a critical mediator of therapeutic resistance across multiple cancer treatment modalities, including chemotherapy, targeted therapy, radiotherapy, and immunotherapy. Its dynamic and reversible nature provides cancer cells with a remarkable plasticity to rapidly adapt and survive under therapeutic pressure (82, 83). Rather than operating through a single mechanism, the m^6^A machinery orchestrates a coordinated multi-faceted response that drives resistance. As summarized in **Figure 3**, the m^6^A regulators promote tumor cell resistance through distinct yet interconnected molecular programs. These include enhancing DNA damage repair to overcome genotoxic stress, upregulating drug efflux pumps and pro-survival signaling to create a multidrug-resistant phenotype and sculpting an immunosuppressive tumor microenvironment to evade immune surveillance (84–86). Specifically, m^6^A regulators promote tumor cell resistance to radiation via stabilizing mRNA, regulating HR and inhibiting signaling pathways. In the context of immunotherapy, m^6^A regulators promote the stability of target gene mRNA and affect the recruitment of immune cells and the secretion of cytokines. Furthermore, various pathways of m^6^A regulators drive resistance to chemical drugs (Cisplatin, Gemcitabine) and kinase inhibitors (83, 87). The following sections will detail the molecular mechanisms underpinning each of these epitranscriptome-driven resistance pathways, highlighting how the dysregulation of writers, erasers, and readers enables cancer cell survival and ultimately leads to treatment failure and relapse.

### 5.1 Chemotherapy

Resistance to chemotherapeutic agents is frequently driven by epitranscriptomic modifications. A key mechanism involves the m^6^A-mediated upregulation of drug efflux pumps, such as MDR1/ABCB1, which enhance the expulsion of chemotherapeutic drugs from cancer cells, reducing their intracellular concentration and efficacy (88). Furthermore, the epitranscriptome enhances DNA damage repair capacity, a primary mechanism of resistance to DNA-damaging chemotherapies. METTL3-catalyzed m^6^A modification plays a direct role in the homologous recombination (HR) repair pathway. In response to DNA double-strand breaks (DSBs), ATM phosphorylates and activates METTL3, which localizes to damage sites (89). There, it methylates damage-associated RNAs, facilitating the recruitment of HR repair proteins like RAD51 and promoting efficient DSB repair, thereby allowing cancer cells to survive genotoxic effect (89).

### 5.2 Targeted therapy

Resistance to targeted agents is also orchestrated by RNA modifications. For example, in lung cancer, the m^6^A demethylase ALKBH5 promotes resistance to EGFR tyrosine kinase inhibitors by stabilizing FOXM1 transcripts, a transcription factor involved in cell proliferation and drug resistance (45). Conversely, in leukemia, the demethylase FTO confers resistance to PARP inhibitors. FTO is upregulated by oncogenic drivers like MLL-fusions and PML-RARA, and its activity leads to the demethylation and downregulation of tumor suppressor mRNAs such as ASB2 and RARA (45). This suppression of negative regulators of growth diminishes the efficacy of PARP inhibition, enabling cell survival.

### 5.3 Radiotherapy

The response to radiotherapy is significantly influenced by the epitranscriptome. As with chemotherapy, the METTL3-m^6^A axis is crucial for repairing radiation-induced DNA damage. METTL3-mediated methylation of RNAs at DSB sites is essential for the recruitment of DNA repair machinery. The reader protein YTHDC1 binds these m^6^A-modified RNAs, protecting them from degradation and promoting the formation of DNA-RNA hybrids that facilitate the accumulation of repair factors like DNA polymerase κ (Pol κ) at the damage site (90). This pathway is critical for efficient repair and cell survival post-irradiation. Consequently, inhibition of this mechanism sensitizes cancer cells to radiotherapy.

### 5.4 Immunotherapy

The epitranscriptome is a key determinant of the tumor immune microenvironment and the response to immune checkpoint blockade (ICB). Distinct m^6^A methylation patterns in tumors correlate with different immune phenotypes: immune-inflamed (“hot”), immune-excluded, and immune-desert (“cold”) (91–93). These patterns directly influence the infiltration of immunosuppressive cells. For instance, loss of the demethylase ALKBH5 in melanoma cells alters the m^6^A landscape, leading to reduced expression of MCT4/Slc16a3, a lactate transporter. This reduction decreases lactate secretion into the tumor microenvironment, which in turn impairs the expansion and differentiation of myeloid-derived suppressor cells (MDSCs) and Tregs (93, 94). The resulting shift in the immune contexture-characterized by fewer immunosuppressive cells and increased DC abundance-renders tumors more susceptible to anti-PD-1 therapy (79). Beyond erasers, reader proteins also play a direct role in mediating an immunosuppressive environment. In ovarian cancer, the m^6^A reader YTHDF1 is frequently amplified and overexpressed. YTHDF1 drives tumor progression by enhancing the translation of its target transcripts in an m^6^A-dependent manner. A key oncogenic target of YTHDF1 is the translation initiation factor EIF3C. By creating a feed-forward loop that augments the global translational capacity of cancer cells, the YTHDF1-EIF3C axis promotes a highly aggressive and immunosuppressive tumor phenotype, contributing to therapy resistance (95). This demonstrates that targeting m^6^A regulators can effectively modulate the TME and overcome resistance to immunotherapy.

## 6 Therapeutic targeting of the epitranscriptome

The dynamic and reversible nature of RNA modifications, particularly m^6^A, presents a promising and novel therapeutic avenue for cancer treatment. However, the context-dependent roles of its regulators necessitate a targeted, subtype-specific approach. The pharmacological toolkit for manipulating the epitranscriptome has expanded rapidly, as summarized in **Supplementary Table S2**, with numerous companies advancing candidates against writers, erasers, and readers. The critical next step is to align these therapeutic modalities with the m^6^A-based taxonomy we propose. The efficacy of a given inhibitor is not universal but is intrinsically linked to the dominant m^6^A subtype of the tumor.

Targeting the writers, erasers, and readers of the epitranscriptome with small molecules, RNA-based therapies, or rational combination strategies offers a powerful approach to disrupt the oncogenic pathways that are fundamentally dependent on this regulatory layer. The development of these agents signifies a paradigm shift in oncology, moving beyond traditional DNA and protein targets to directly modulate the RNA regulatory code itself. This section details the current and emerging arsenal of therapeutic strategies, from pioneering small-molecule inhibitors that validate the druggability of these proteins to next-generation modalities that promise greater potency and specificity.

### 6.1 First-generation catalytic inhibitors

The most advanced area of epitranscriptomic therapeutics is the development of small-molecule inhibitors that competitively target the catalytic sites of writers and erasers, thereby directly altering the cellular m^6^A landscape. For the m^6^A eraser FTO, significant progress has been made through a combination of drug repurposing and structure-based design. The anti-inflammatory drug meclofenamic acid (MA) was identified as an FTO inhibitor that increases global cellular m^6^A levels (79). This discovery paved the way for more potent and selective derivatives; notably, FB23 and its optimized counterpart, FB23-2, a benzohydroxamic acid derivative, exhibit dramatically improved inhibitory activity (IC50 = 0.06 μM for FB23) and enhanced cellular uptake (13). FB23–2 binds directly within FTO’s substrate-binding pocket, as confirmed by co-crystal structures, and demonstrates potent anti-leukemic effects both *in vitro* and *in vivo*. Its mechanism of action involves increasing m^6^A methylation on key tumor suppressor transcripts such as ASB2 and RARA, leading to their stabilization and the subsequent suppression of oncogenic drivers like MYC and CEBPA (45, 48). In a similar vein, the oncometabolite R-2-hydroxyglutarate (R-2HG), which accumulates in IDH-mutant cancers, has been shown to function as an FTO inhibitor, contributing significantly to its anti-tumor effects in AML models (48). Furthermore, natural compounds like Rhein have also been identified as FTO inhibitors, demonstrating potential in suppressing cancer progression (96).

Concurrently, parallel efforts have focused on inhibiting the m^6^A writer complex. A landmark achievement in this area was the development of STM2457, a highly potent (IC50 = 16.9 nM) and selective METTL3 inhibitor discovered through high-throughput screening and subsequent optimization (75, 97). STM2457 functions by competitively binding the S-adenosylmethionine (SAM) binding site of the METTL3-METTL14 heterodimer, the catalytic core of the writer complex. In models of acute myeloid leukemia, treatment with STM2457 phenocopies the genetic ablation of METTL3, robustly impairing leukemia cell proliferation and clonogenic growth, while inducing differentiation and apoptosis. These anti-tumor effects are driven by a reduction in the protein levels of key METTL3 oncogenic targets, such as SP1 and BRD4, without adversely affecting normal hematopoiesis, highlighting a potential therapeutic window (98). The compelling pre-clinical data generated by these first-generation inhibitors, STM2457 and FB23-2, provide unequivocal proof-of-concept that the m^6^A epitranscriptomic machinery is druggable and represents a viable target for cancer therapy. The rapid expansion of this pharmacological toolkit is illustrated in **Supplementary Table S3**, which summarizes a wide array of additional small-molecule inhibitors and activators targeting writers, erasers, and readers. These compounds, ranging from repurposed drugs like Entacapone to naturally derived molecules like Quercetin and specialized research tools like the YTHDF2 inhibitor DF-A7, have been instrumental in dissecting the complex biological functions of m^6^A modifiers in various cancer models. While STM2457 and FB23–2 represent the most advanced and specific candidates, the diverse agents highlight the intense research efforts and the potential for discovering new chemical scaffolds for clinical development.

### 6.2 Next-generation therapeutic modalities

While catalytic inhibitors have successfully validated the druggability of the m^6^A machinery, the field is rapidly advancing towards more sophisticated and potentially more powerful therapeutic modalities. These next-generation approaches aim to overcome limitations of traditional occupancy-based inhibitors, such as the need for sustained high target occupancy and vulnerability to resistance mutations, while also expanding the scope of targetable proteins within the epitranscriptome.

One of the most promising emerging modalities is the use of Proteolysis Targeting Chimeras (PROTACs). These heterobifunctional molecules are designed to recruit an E3 ubiquitin ligase to a protein of interest, leading to its ubiquitination and subsequent degradation by the proteasome. A PROTAC designed to degrade a key m^6^A regulator, such as METTL3 or FTO, would offer several potential advantages over catalytic inhibition. Its event-driven mechanism of action is catalytic, meaning a single PROTAC molecule can facilitate the degradation of multiple target proteins, potentially leading to a more potent and sustained pharmacological effect (99). Furthermore, by removing the target protein entirely, a PROTAC could overcome resistance mechanisms related to protein overexpression or point mutations that reduce drug-binding affinity. Although m^6^A-targeted PROTACs are still in early stages of development, the broader PROTAC platform has been clinically validated for other oncogenic targets. As summarized in **Table 3**, numerous PROTACs are now in clinical trials, targeting proteins such as the androgen and estrogen receptors (AR, ER), and Bruton’s tyrosine kinase (BTK). The rapid progression of these agents underscores the feasibility of targeted protein degradation and paves the way for applying this powerful modality to the m^6^A epitranscriptomic machinery (100, 101).

**Table 3:** Selected PROTAC degraders in clinical development for oncology.

Degrader	Target	E3 ligase	Key indications	NCT number	Phase	Status (as of May 2024)	Company/sponsor	ROA
Hormone receptors								
ARV-110 (Bavdegalutamide)	AR	CRBN	mCRPC	NCT03888612	II	Active, Recruiting	Arvinas	Oral
CC-94676	AR	CRBN	mCRPC	NCT04428788	I	Active, Recruiting	Bristol Myers Squibb	Oral
ARV-766	AR	Undisclosed	mCRPC	NCT05067140	I/II	Active, Recruiting	Arvinas	Oral
AC176	AR	Undisclosed	Prostate Cancer	NCT05241613	I	Active, Recruiting	Accutar Biotech	Oral
HP518	AR	Undisclosed	mCRPC	NCT05252364	I	Active, Recruiting	Hinova Pharmaceuticals	Oral
ARV-471 (Vepdegestrant)	ER	CRBN	Breast Cancer	NCT04072952	II	Active, Recruiting	Arvinas/Pfizer	Oral
AC682	ER	CRBN	Breast Cancer	NCT05080842	I	Active, Recruiting	Accutar Biotech	Oral
Kinases & signaling								
NX-5948	BTK	CRBN	B-cell Malignancies, Autoimmune Disease	NCT05131022	I	Active, Recruiting	Nurix Therapeutics	Oral
NX-2127	BTK	CRBN	B-cell Malignancies	NCT04830137	I	Active, Recruiting	Nurix Therapeutics	Oral
BGB-16673	BTK	Undisclosed	B-cell Malignancies	NCT05006716	I	Active, Recruiting	BeiGene	Oral
HSK29116	BTK	Undisclosed	B-cell Malignancies	NCT04861779	I	Active, Recruiting	Haisco Pharmaceutical	Oral
ASP3082	KRAS G12D	Undisclosed	Solid Tumors (e.g., CRC, Pancreatic)	NCT05382559	I	Active, Recruiting	Astellas Pharma	IV
KT-333	STAT3	Undisclosed	Lymphomas, Solid Tumors	NCT05225584	I	Active, Recruiting	Kymera Therapeutics	IV
KT-413	IRAK4	CRBN	MYD88-mutant DLBCL	NCT05233033	I	Active, Recruiting	Kymera Therapeutics	IV
Epigenetic regulators								
FHD-609	BRD9	Undisclosed	Synovial Sarcoma	NCT04965753	I	Active, Recruiting	Foghorn Therapeutics	IV
CFT8634	BRD9	CRBN	Synovial Sarcoma	NCT05355753	I/II	Active, Recruiting	C4 Therapeutics	Oral
Anti-apoptotic & other								
DT2216	BCL-XL	VHL	Solid Tumors, Lymphomas	NCT04886622	I	Active, Recruiting	Dialectic Therapeutics	IV
Other targets								
CFT8919	EGFR L858R	CRBN	NSCLC	NCT05686985	I	Active, Recruiting	C4 Therapeutics	Oral

This table illustrates the rapid clinical translation of the PROTAC platform, highlighting targets relevant to the discussion of next-generation epitranscriptomic therapeutics. (AR, Androgen Receptor; BC, Breast Cancer; CRBN, Cereblon; CRC, Colorectal Cancer; DLBCL, Diffuse Large B-Cell Lymphoma; ER, Estrogen Receptor; mCRPC, Metastatic Castration-Resistant Prostate Cancer; NSCLC, Non-Small Cell Lung Cancer; ROA, Route of Administration; VHL, von Hippel-Lindau).

Another frontier is the direct targeting of reader proteins, which have historically been considered challenging due to their protein-RNA interaction interfaces. However, readers like the IGF2BP family are critical nodes in oncogenesis, stabilizing a network of potent oncogenic mRNAs, including MYC. Small molecules that specifically disrupt the interaction between a reader like IGF2BP1 and its target oncogenic transcripts could offer a highly precise therapeutic strategy. Such an approach would selectively downregulate key drivers of tumorigenesis without altering the global m^6^A landscape, potentially mitigating on-target toxicities associated with writer or eraser inhibition that affect thousands of transcripts (41, 90). Advances in screening and structural biology are making this challenging goal increasingly feasible.

Finally, RNA-based therapies continue to be a vital tool and potential therapeutic avenue. siRNA and shRNA-mediated knockdown of specific writers (METTL3, METTL14), erasers (FTO, ALKBH5), or readers (YTHDF1/2, IGF2BP1) has been extensively and successfully used in pre-clinical models to validate their oncogenic functions, inducing anti-tumor effects such as differentiation, apoptosis, and impaired self-renewal (13, 44, 45, 102). For instance, ALKBH5 knockdown in melanoma cells was shown to alter the tumor immune microenvironment and enhance the efficacy of anti-PD-1 therapy (79). The primary hurdle for this modality remains the development of efficient, safe, and tumor-specific systems for the *in vivo* delivery of these RNAi agents.

### 6.3 Rational combination therapy: a logic framework

The m^6^A epitranscriptome does not operate in isolation; it functions as a central signaling hub that cancer cells co-opt to develop resistance to a wide array of therapies. This biological understanding provides a powerful rationale for combining m^6^A-targeted agents with established standard-of-care treatments. Rather than applying combinations empirically, the choice of regimen should be guided by the specific resistance mechanism driven by the dysregulated m^6^A regulator in each cancer context, creating a logical and personalized framework for combination therapy.

The rationale for combining m^6^A-targeted agents with chemotherapy is strongly supported by mechanistic studies. For example, FTO inhibitors like FB23–2 and R-2HG can reverse resistance to PARP inhibitors and tyrosine kinase inhibitors in AML. They achieve this by preventing the FTO-mediated demethylation and destabilization of tumor suppressor transcripts such as ASB2 and RARA, thereby restoring the cellular checks and balances that the targeted therapies rely upon (53, 83). Conversely, METTL3 inhibitors can sensitize cancer cells to DNA-damaging chemotherapies. This is because METTL3-catalyzed m^6^A modification plays a direct role in the homologous recombination (HR) repair pathway; inhibiting METTL3 impairs the recruitment of repair proteins like RAD51 to DNA damage sites, compromising the cancer cell’s ability to survive genotoxic stress (89).

The combination with radiotherapy follows a similar logic, targeting the epitranscriptome’s role in DNA damage response. The METTL3-m^6^A-YTHDC1 axis has been identified as critical for repairing radiation-induced DNA double-strand breaks. Inhibiting this axis, particularly through METTL3 inhibition, sensitizes cancer cells to radiotherapy by impairing the rapid, RNA methylation-dependent recruitment of DNA repair factors, such as DNA polymerase κ, to the damage sites (49, 103).

Perhaps one of the most exciting applications is the combination of m^6^A-targeted agents with immunotherapy. The epitranscriptome is a master regulator of the tumor immune microenvironment and targeting it can convert “cold” tumors into “hot” ones. For instance, inhibiting the eraser ALKBH5 in melanoma cells reduces lactate secretion, which in turn impairs the expansion and function of immunosuppressive myeloid-derived suppressor cells (MDSCs) and regulatory T cells (Tregs), thereby enhancing the response to anti-PD-1 therapy (104). Similarly, depleting the reader YTHDF1 in dendritic cells enhances cross-presentation of tumor antigens to T cells and synergizes with PD-L1 blockade (58). Additionally, FTO inhibition has been shown to reduce the stability of key immunosuppressive checkpoint transcripts, including PD-1, CTLA-4, and LILRB4, in immune cells, potentially enhancing anti-tumor immunity and the efficacy of checkpoint blockade (105, 106).

In conclusion, the development of highly specific small-molecule inhibitors such as STM2457 and FB23–2 has provided the foundational tools to pharmacologically manipulate the epitranscriptome. The ongoing clinical evaluation of next-generation inhibitors like STC-15, an oral derivative of STM2457 (NCT05584111), represents a pivotal step in translating this novel therapeutic paradigm from the laboratory to the clinic. By leveraging a deep understanding of the mechanistic links between specific m^6^A regulators and distinct therapy resistance pathways, the future of cancer treatment will likely involve rational combinations that include epitranscriptome-targeting agents to overcome the formidable challenge of therapeutic resistance and improve patient outcomes.

## 7 Clinical translation: an m^6^a-based taxonomy and therapeutic roadmap

The immense promise of targeting the m^6^A epitranscriptome is tempered by the significant challenge of context-dependency, where the same regulator can exert opposing roles in different cancer types or stages. To bridge this gap between mechanistic understanding and clinical application, we propose a move from a descriptive model to a predictive, subtype-driven framework. By integrating pan-cancer analyses from gastric cancer (STAD), low-grade glioma (LGG), acute myeloid leukemia, and others, we can define distinct m^6^A-driven subtypes and create a diagnostic-therapeutic roadmap for personalized oncology (107, 108).

### 7.1 Decoding context-dependency: defining m^6^A subtypes

The seemingly paradoxical roles of m^6^A regulators across different cancers reflect distinct biological states rather than experimental noise. To reconcile these context-dependent functions and enable clinical translation, emerging pan-cancer evidence supports the classification of tumors based on their predominant dysregulated m^6^A component.

The feasibility and clinical utility of m^6^A-based tumor classification is substantiated by comprehensive pan-cancer studies. A landmark analysis by Li et al. systematically characterized the genetic and expression landscapes of 20 m^6^A regulators across 33 cancer types in The Cancer Genome Atlas (TCGA) (107). This study revealed that m6A regulators exhibit cancer-specific patterns of copy number variation (CNV) and differential expression, with clear prognostic associations. For instance, readers such as IGF2BP1/2/3 and YTHDF1/3 showed frequent CNV amplification and corresponding overexpression in multiple cancers, whereas writers like METTL14 and erasers like ALKBH5 often exhibited reduced expression. Critically, unsupervised clustering based solely on m^6^A regulator expression patterns successfully stratified patients into subgroups with distinct clinical outcomes. In kidney cancer, this approach identified two molecular subgroups-an m^6^A regulator-high (RM-high) group and an RM-low group with significantly different overall survival (log-rank p = 0.005) (107). This pan-cancer analysis provides fundamental proof-of-concept: coordinated dysregulation of the m^6^A machinery defines biologically coherent tumor subtypes with inherent prognostic value.

The prognostic power and biological coherence of these m^6^A-based subtypes are further validated by in-depth mechanistic studies in specific cancers, which align precisely with our proposed taxonomy. These studies demonstrate that dysregulation of specific regulator classes drives coherent oncogenic programs. For example, work in ovarian cancer provides a clear archetype for the Reader-Amplified Subtype. Liu et al. showed that the m^6^A reader YTHDF1 is frequently amplified and overexpressed in high-grade serous ovarian carcinoma, with its high expression correlating strongly with advanced FIGO stage and shorter overall and progression-free survival (109). Functionally, YTHDF1 acts as a potent oncogene, promoting tumor cell proliferation, migration, and invasion both *in vitro* and *in vivo* by enhancing the translation of key targets like the translation initiation factor EIF3C in an m^6^A-dependent manner. Similarly, the Eraser-High Subtype is exemplified by studies in glioblastoma, where Zhang et al. demonstrated that the demethylase ALKBH5 is essential for maintaining the tumorigenicity of glioblastoma stem-like cells (GSCs) by sustaining FOXM1 expression through transcript demethylation (63). This establishes a direct link between eraser dysregulation, stemness maintenance, and therapeutic resistance.

Building upon this converging evidence, we propose a molecular taxonomy that classifies tumors into four major m^6^A-driven subtypes based on their dominant dysregulated component. Each subtype is defined by specific molecular and functional criteria, supported by extensive evidence of component-specific dysregulation across cancer types, including regulation of both coding and non-coding RNAs (107).

The Writer-Dominant (METTL3/14-High) Subtype is characterized by overexpression of core methyltransferase components and elevated global m^6^A deposition. In AML, METTL3 is identified as a dependency gene essential for leukemia cell survival and proliferation, with its loss reversing differentiation blocks and promoting apoptosis (110). In HCC, METTL3 is significantly elevated and stabilizes oncogenic lncRNAs such as RMRP to activate the TGFBR1/Smad2/Smad3 pathway (111). METTL3 also modifies lncRNA ANRIL in pancreatic cancer to enhance gemcitabine resistance via enhanced DNA repair (112). METTL14 shows similar oncogenic roles, stabilizing lncRNA FAM225A in nasopharyngeal carcinoma to promote metastasis (113). Other writers include WTAP, which stabilizes lncRNA FOXD2-AS1 in osteosarcoma (114), and KIAA1429, which regulates circDLC1 in HCC (115). Assignment criteria for this subtype include: (1) METTL3/METTL14 mRNA or protein expression significantly higher in acute myeloid leukemia (AML) cells compared to normal hematopoietic stem and progenitor cells (HSPCs); (2) High METTL3 dependency score in CRISPR screens; (3) Enrichment of m^6^A modifications on oncogenic transcripts like MYC, BCL2, and SP1/2; and (4) Evidence of writer-mediated regulation of key ncRNAs such as lncRNA RMRP or circDLC1.

FTO is highly expressed in specific AML subtypes (MLL-rearranged, PML-RARA, FLT3-ITD, NPM1-mutant) and promotes leukemogenesis by destabilizing tumor suppressor mRNAs like ASB2 and RARA (44, 76). In glioblastoma, ALKBH5 is aberrantly upregulated in GSCs and its depletion inhibits self-renewal and tumorigenesis (63). In breast cancer, hypoxia-induced ALKBH5 upregulation enriches cancer stem cells through NANOG stabilization (116). Beyond mRNA targets, FTO removes m^6^A marks from lncRNAs including LINC00022 in esophageal squamous cell carcinoma (ESCC), preventing YTHDF2-mediated degradation and promoting proliferation (117). ALKBH5 is aberrantly upregulated in glioblastoma stem cells where it stabilizes lncRNA NEAT1 under hypoxic conditions to upregulate CXCL8/IL8 (118). In pancreatic cancer, ALKBH5 regulates lncRNA KCNK15-AS1 to influence invasion and metastasis (119). Assignment criteria include: (1) FTO or ALKBH5 expression in the top tertile of tumor cohorts; (2) Correlation with hypoxic signatures (for ALKBH5); and (3) Stabilization of known target transcripts (MYC/CEBPA for FTO; FOXM1/NANOG for ALKBH5).

The Reader-Amplified (IGF2BP-High/YTHDF1-High) Subtype features amplification or overexpression of reader proteins. IGF2BP1/2/3 are unfavorable prognostic factors in gastric cancer (STAD) and drive proliferation and glycolysis (120). YTHDF1 is frequently amplified in ovarian cancer and promotes an immunosuppressive phenotype via EIF3C translation (109). IGF2BP3 stabilizes oncogenic mRNAs like MYC through m^6^A recognition (13). IGF2BP1 stabilizes circMDK in HCC to promote proliferation and invasion (121). IGF2BP2 stabilizes lncRNA DANCR in prostate cancer (122). IGF2BP3 stabilizes lncRNA KCNMB2-AS1 in cervical cancer (123). YTHDF1 promotes translation of lncRNA-encoded micropeptides in ESCC (109, 124). YTHDF2 stabilizes lncRNA KCNQ1OT1 in laryngeal squamous cell carcinoma (125). YTHDC1 promotes circRNA biosynthesis and export in HCC (126). Assignment criteria include: (1) IGF2BP or YTHDF family gene amplification or protein overexpression (e.g., by IHC or RNA-seq); (2) Co-upregulation of validated target oncogenes (MYC, LIN28B, SOX2); (3) Association with poor prognosis in clinical cohorts; and (4) Evidence of reader-mediated stabilization or degradation of key ncRNAs such as circMDK or lncRNA DANCR.

The Immune-Modulatory Subtype is identified through clustering analyses that reveal distinct m^6^A-immune phenotypes. In STAD, an “immune-inflamed” m^6^A cluster (Cluster C) shows high CD8^+^ T cell and dendritic cell infiltration (127). In LGG, clusters with high M2 macrophage and neutrophil infiltration correlate with poor outcomes (128). Loss of ALKBH5 creates “hot” tumors in melanoma models (129). METTL14-mediated m^6^A modification of lncRNA MIR155HG in HCC regulates PD-L1 expression (130). ALKBH5 regulates lncRNA NEAT1 to influence CXCL8/IL8 expression in glioblastoma (118). Assignment criteria include: (1) Distinct m^6^A regulator expression pattern by unsupervised clustering; (2) Specific immune cell infiltration signature; (3) Enrichment of m^6^A modifications on immune checkpoint transcripts.

Regarding multi-subtype tumors and hybrid features, biological complexity dictates that tumors may not exclusively fit a single subtype but can exhibit molecular features of multiple m^6^A-driven categories. This is strongly supported by genomic and functional evidence of co-dysregulation across different classes of m^6^A regulators. For instance, pan-cancer analyses reveal that genetic alterations and expression changes in writers, erasers, and readers frequently co-occur, and their expression is highly correlated across cancer types, indicating intertwined regulatory networks (107). A prime functional example is found in Acute Myeloid Leukemia, where a tumor can simultaneously display characteristics of both Writer-Dominant and Reader-Amplified subtypes. The writer METTL3 is recruited to specific promoters (SP1/SP2) to install m6A marks that are critical for the efficient translation of these transcripts, which in turn drive MYC expression-a hallmark of the Writer-Dominant program (44). Concurrently, the reader IGF2BP2 (and other IGF2BPs) can recognize and stabilize m^6^A-modified MYC mRNA, amplifying its oncogenic output and exemplifying a Reader-Amplified mechanism (13, 48). Furthermore, the co-occurrence of high writer (METTL3) and high eraser activity has been documented in specific AML subtypes, such as those with MLL rearrangements or NPM1 mutations, where FTO-mediated demethylation of tumor suppressors like ASB2 and RARA operates alongside METTL3-driven oncogene translation (45). This functional overlap underscores that the m^6^A machinery operates as a coordinated network rather than in isolated silos. Therefore, for clinical subtyping, when a tumor meets molecular criteria for multiple categories, the dominant subtype is assigned based on the highest regulator expression level and functional dependency score. However, the identification of secondary hybrid features is critically important, as it provides a direct rationale for designing rational combination therapies that simultaneously target multiple dysregulated axes.

### 7.2 A diagnostic and decision-making roadmap

To translate the proposed m^6^A-based taxonomic framework into clinical practice, we propose a comprehensive diagnostic-therapeutic roadmap that integrates liquid biopsy-based biomarker detection with subtype-directed treatment strategies. This roadmap is structured as a continuous clinical workflow that begins with patient presentation and extends through longitudinal monitoring, with clear decision points informed by the molecular criteria established in Section 7.1. The process initiates with Patient Presentation and Liquid Biopsy Acquisition at the time of cancer diagnosis or disease progression, where a peripheral blood sample is collected for liquid biopsy analysis. This minimally invasive approach enables the isolation of circulating tumor biomarkers, including circulating tumor cells (CTCs), cell-free RNA (cfRNA), and exosomes. The choice of analyte is context-dependent: cfRNA offers direct epitranscriptomic profiling but presents technical challenges due to its lability and susceptibility to degradation by RNases; exosomal proteins provide more stable detection of m^6^A regulators such as METTL3 or YTHDF1 but offer indirect functional information regarding the actual m^6^A modification landscape (131–134). This initial step facilitates real-time molecular profiling without the need for repeated invasive tissue biopsies, aligning with the principles of dynamic cancer monitoring and personalized oncology.

The next phase, m^6^A Subtyping via Multi-Omics Profiling, involves comprehensive molecular analysis to assign the tumor to one of the four proposed subtypes. The sample undergoes targeted RNA sequencing (RNA-seq) to quantify expression levels of key m^6^A regulators-writers (METTL3, METTL14, WTAP), erasers (FTO, ALKBH5), and readers (IGF2BP1-3, YTHDF1-3). Complementarily, mass spectrometry (LC-MS/MS) or immunoaffinity-based methods (MeRIP-seq, m^6^A-qPCR) measure global m^6^A levels and specific modification patterns on oncogenic and immune-related transcripts such as MYC, PD-L1, and CXCL10 (135, 136). Subtype assignment follows a predefined decision algorithm based on evidence-based criteria: tumors with significant METTL3/METTL14 overexpression and elevated global m^6^A are classified as Writer-Dominant; those with high FTO or ALKBH5 expression and global hypomethylation as Eraser-High; tumors with reader amplification and corresponding oncogene stabilization as Reader-Amplified; and those with distinct m^6^A-immune gene signatures as Immune-Modulatory. For tumors meeting criteria for multiple subtypes-a possibility due to biological complexity-the primary subtype is assigned based on the highest regulator expression and functional dependency, while secondary characteristics are documented to inform potential combination therapy.

Following subtype classification, Assignment to Rational Combination Therapy directs patients to subtype-specific therapeutic regimens. Writer-Dominant tumors are candidates for METTL3 catalytic inhibitors such as STM2457 or its oral derivative STC-15, strategically combined with DNA-damaging chemotherapy or radiotherapy to exploit synthetic lethality given the writer’s role in homologous recombination repair (75, 89, 137). Eraser-High tumors receive FTO inhibitors (FB23-2) or ALKBH5 inhibitors combined with immune checkpoint blockade (e.g., anti-PD-1/PD-L1) to reverse immunosuppression and enhance T-cell activity, as demonstrated in melanoma and AML models (79, 104–106). Reader-Amplified tumors are directed toward emerging reader-targeted therapies, such as small molecules disrupting the IGF2BP-MYC interaction or YTHDF1 inhibitors, potentially combined with pathway-specific agents (57, 138). Immune-Modulatory tumors are treated with immunotherapy regimens selected based on their specific m^6^A-immune phenotype, with potential priming using epitranscriptomic modulators like ALKBH5 inhibitors to convert immunologically “cold” tumors to “hot” ones (139–141). For tumors with hybrid features-such as those with both Writer-Dominant and Reader-Amplified characteristics-combination strategies targeting both axes (METTL3 inhibitor plus IGF2BP inhibitor) are considered within clinical trial frameworks or off-label use were supported by safety data.

The final component, Longitudinal Monitoring via Dynamic m^6^A Biomarkers, implements serial liquid biopsies to assess treatment response and detect emerging resistance. During therapy, repeat blood samples track quantitative changes in m^6^A regulator expression, global and transcript-specific m^6^A levels, and subtype signature persistence or evolution. For example, a decline in METTL3 cfRNA or an increase in m^6^A on MYC mRNA may indicate effective METTL3 inhibition, while rising exosomal FTO levels could signal developing resistance to demethylase-targeted therapy (55, 131). This real-time molecular monitoring enables adaptive treatment adjustments, such as switching therapies or adding combination agents based on evolving molecular profiles. The non-invasive nature of liquid biopsy and the dynamic responsiveness of m^6^A modifications make this an ideal platform for implementing precision oncology in a clinically feasible manner, moving beyond static tissue-based diagnostics to a continuous, biomarker-guided treatment paradigm that can address tumor evolution and therapeutic resistance (142, 143).

## 8 Liquid biopsies for m^6^A-based diagnosis and monitoring: from cfRNA to exosomal proteins

The transition of m^6^A research from a mechanistic understanding to clinical application hinges on the development of robust, non-invasive diagnostic tools. Liquid biopsy, which analyzes biomarkers in body fluids like blood, represents a paradigm shift in oncology, enabling real-time monitoring of tumor dynamics, early detection, and assessment of therapeutic response without the need for invasive tissue sampling (132, 133). The epitranscriptome, with its dynamic and cancer-specific alterations, is poised to be a cornerstone of this new diagnostic frontier. This section explores the technical landscape, specific biomarker candidates, and the immense potential of integrating m^6^A analysis into liquid biopsy platforms for transforming cancer management.

### 8.1 Technical challenges: cfRNA vs. exosomal proteins

A central consideration in developing m^6^A-based liquid biopsies is the choice of analyte. Two primary sources are cell-free RNA (cfRNA) and exosomal proteins, each with distinct advantages and limitations.

Detecting m^6^A-modified cfRNA offers a direct window into the epitranscriptomic state of tumors. cfRNA, released into the bloodstream through apoptosis, necrosis, or active secretion (134, 135), can reflect the real-time transcriptional and post-transcriptional regulation within tumor cells. Studies on non-small cell lung cancer (NSCLC) and other malignancies have demonstrated that cfRNA sequencing (cfRNA-Seq) can distinguish cancer patients from healthy individuals by revealing disease-specific gene expression signatures, immune repertoire (TCR/BCR) signals, and even microbial-derived transcripts (136, 144). The primary advantage of this approach is the direct measurement of the modification itself. For instance, techniques like MeRIP-seq could, in principle, be adapted to profile m^6^A epitranscriptomes from circulating RNA, allowing for the detection of global m^6^A shifts or hypermethylation of specific oncogenic transcripts like MYC or *PD-L1* in patient plasma. However, the profound technical challenge is the inherent lability and low abundance of cfRNA. RNA is susceptible to degradation by ubiquitous RNases, leading to short fragment lengths and a low overall concentration in circulation. This makes the reliable detection of specific m^6^A-modified transcripts technically demanding and requires highly sensitive, specialized protocols to preserve RNA integrity and avoid artifacts.

In contrast, measuring m^6^A regulator proteins (e.g., METTL3, FTO, YTHDF1) packaged within exosomes presents a more stable alternative. Exosomes are lipid-bilayer vesicles actively secreted by cells that protect their cargo from degradation. They are abundant in blood and contain proteins, lipids, and nucleic acids reflective of their cell of origin. The key advantage here is stability; exosomal proteins are far less susceptible to degradation than naked cfRNA, making them more reliable analytes for clinical assays. Furthermore, the detection of these proteins, potentially via immunoassays or proximity extension assays, is often more technically straightforward and amenable to high-throughput clinical workflows than RNA modification mapping. For example, the overexpression of METTL3 or YTHDF1 in tumor tissues could be mirrored by their elevated levels in circulating exosomes, serving as a surrogate, stable biomarker for an “m^6^A-high” tumor subtype. The limitation of this approach is its indirect nature. Measuring protein levels does not directly report on the functional outcome-the m^6^A modification landscape-which can be influenced by complex regulatory dynamics. A tumor might overexpress METTL3 protein, but its catalytic activity could be contextually modulated. Therefore, while exosomal proteins offer a robust and practical detection method, they may not capture the full functional complexity of the epitranscriptome. The choice between these analytes may ultimately be context-dependent, with a combination of both providing the most comprehensive picture. For early detection and prognosis, stable exosomal proteins might be preferable, while for monitoring dynamic response to therapy, direct measurement of m^6^A on cfRNA could be more informative.

### 8.2 Promising biomarker candidates

Substantial research has identified specific components of the m^6^A machinery as promising biomarker candidates for various clinical applications, from early detection to monitoring therapeutic response.

For Early Detection: Global m^6^A Levels and Specific Regulator Profiles. Pan-cancer studies suggest that the global abundance of m^6^A modifications in tissue can be a distinguishing feature, with certain cancers exhibiting widespread hypomethylation (107). This global signature could potentially be detected in blood-based assays. For instance, mass spectrometry-based quantification of m^6^A nucleosides in circulating RNA could serve as a pan-cancer screening tool. More specifically, the detection of exosomal proteins or cfRNA transcripts for specific m^6^A regulators shows great promise. In glioblastoma, the detection of the FTO inhibitor FTO-04 in circulation could indicate the presence of FTO-driven tumors (145). Similarly, the upregulation of m^6^A-related genes in pancreatic cancer, such as METTL3 and IGF2BP2, could form the basis for an early detection signature, especially in high-risk populations.

For Prognosis and Subtyping: Reader and Writer Amplification. The amplification and overexpression of specific “reader” and “writer” proteins are strongly correlated with aggressive disease and poor prognosis, making them excellent prognostic biomarkers. The reader YTHDF1 is a prime example. Its frequent amplification and overexpression in ovarian cancer drives a highly aggressive and immunosuppressive phenotype (146). Detecting YTHDF1 protein in exosomes or its transcript in cfRNA could identify patients with particularly virulent disease, guiding more aggressive initial therapy. Similarly, in stomach adenocarcinoma (STAD), a high expression of IGF2BP1/2/3 in tumors are unfavorable prognostic factor, and an m^6^A-based signature (m^6^ASig) derived from these and other regulators can stratify patients into distinct risk groups. These m^6^A-based subtypes, such as the “Reader-Amplified” or “Writer-Dominant” classifications, have direct prognostic implications and can be translated into a liquid biopsy format by building multi-analyte panels that quantify the key regulators defining each subtype.

For Monitoring Therapeutic Response: Dynamic m^6^A Changes on Key Transcripts. Perhaps the most powerful application of m^6^A liquid biopsies is the real-time monitoring of treatment efficacy and the emergence of resistance. The dynamic and reversible nature of m^6^A makes it an ideal responsive biomarker. For example, the efficacy of a METTL3 inhibitor like STM2457 could be monitored by tracking the reduction in m^6^A methylation on known target transcripts, such as MYC or BRD4, in cfRNA post-treatment (98). Conversely, the development of resistance to anti-PD-1 immunotherapy has been linked to specific epitranscriptomic alterations. The demethylase ALKBH5 can promote resistance by stabilizing immunosuppressive transcripts. Therefore, a decrease in m^6^A levels on the PD-L1 transcript or an increase in ALKBH5 cfRNA could serve as an early liquid biomarker of immunotherapy failure, allowing for a timely switch to alternative treatments. This principle extends to chemotherapy; the upregulation of FTO in AML relapse samples contributes to chemoresistance, and its detection in plasma could signal impending relapse long before it is clinically apparent (55).

In conclusion, the integration of m^6^A analysis into liquid biopsy platforms is poised to revolutionize cancer diagnostics. By tackling the technical challenges of cfRNA analysis and leveraging the stability of exosomal proteins, clinically viable assays can be developed. These assays will utilize a growing arsenal of m^6^A-based biomarkers-from global levels to specific regulator proteins and modification sites on key transcripts-to enable early detection, refine prognostic stratification, and provide an unprecedented, dynamic view of treatment response, ultimately paving the way for truly personalized epitranscriptome-guided therapy.

## 9 Conclusion

The epitranscriptome has been established as a master regulatory layer governing cancer biology, from driving malignant transformation to mediating therapeutic failure. The development of specific inhibitors against writers and erasers provides compelling proof that this regulatory axis is druggable. However, as this review has argued, the true clinical potential of this paradigm shift lies in navigating its profound context-dependency. The m^6^A-based taxonomic framework proposed herein-classifying tumors into Writer-Dominant, Eraser-High, Reader-Amplified, and Immune-Modulatory subtypes provides a strategic blueprint for personalizing therapy. This model offers a coherent logic to reconcile contradictory findings and directly links tumor biology to specific therapeutic vulnerabilities. Coupled with the integration of m^6^A analysis into liquid biopsies, this framework promises to revolutionize diagnostics by enabling real-time, dynamic subtyping and monitoring of treatment response. While significant translational hurdles remain, a concerted effort to validate these subtypes and their associated biomarkers in clinical cohorts will be essential. The future of epitranscriptome-targeted therapy is not merely in inhibiting a single protein, but in leveraging this deep mechanistic understanding to reprogram the malignant identity of cancer cells through rational, biomarker-guided combinations tailored to the tumor’s dominant m^6^A state.
