# Supplementary material for: Rewriting the RNA code: an m6a-centric framework to classify tumors and guide combination therapies
Source: Front Immunol. 2026 Jan 30;17:1749911. doi: 10.3389/fimmu.2026.1749911 (PMC12901410; doi:10.3389/fimmu.2026.1749911)
Supplement: Supplementary file 1 [file Table1.docx]

**Supplementary Tables**

**Table S1: Regulation of Cancer Metabolism and Therapy Resistance by the m^6^A Epitranscriptome**

| **Cancer Type** | **Regulators** | **Mechanism** | **Functional Outcome** |
| --- | --- | --- | --- |
| **Liver Cancer (HCC)** | YTHDF3 | Inhibits degradation of PFKL mRNA via m^6^A modification | Promote growth and lung metastasis |
|  | ALKBH5 | UBR7 (regulated by ALKBH5) activates Keap1/Nrf2/Bach1/HK2 axis to reduce HK2 | Inhibit glycolysis and proliferation |
|  | METTL14 | Mettl14/USP48/SIRT6 axis inhibits cancer | Inhibit glycolysis |
|  | METTL3, IGF2BP1 | LNCAROD increases PKM2 levels via LNCAROD/PKM2 axis | Induce glycolysis, proliferation, invasion, and resistance to 5-FU |
|  | METTL3 | HBXIP activates glycolytic enzymes via METTL3/HIF-1α axis | Promote glycolysis and malignancy |
|  | ZC3H13 | m6A modification reduces stability of PKM2 mRNA, inhibiting glycolysis | Increase sensitivity to cisplatin chemotherapy |
|  | METTL3, IGF2BP2 | Increase WWP2 expression, promoting glycolysis via WWP2/AKT axis | Induce resistance to doxorubicin chemotherapy |
|  | FTO | LncRNA HOTAIR collaborates with FTO to demethylate HK2 mRNA, increasing its expression. Sevoflurane inhibits this. | Promote glycolysis; Sevoflurane inhibits this effect |
|  | YTHDF1 | CircRHBDD1 recruits YTHDF1 to stabilize PIK3R1 mRNA, activating PI3K/AKT pathway | Promote glycolysis and induce resistance to anti-PD-L1 immunotherapy |
| **Gastric Cancer (GC)** | METTL3, IGF2BP3 | Increase HDGF expression, which binds promoters of GLUT4 and ENO2 | Promote glycolysis, proliferation, and liver metastasis |
|  | METTL3, IGF2BP1 | Increase stability of NDUFA4 mRNA | Improve glycolysis and proliferation |
|  | WTAP | Prolongs half-life of HK2 mRNA | Improve glycolysis and proliferation |
|  | VIRMA | Reduces degradation of LINC00958, promoting GLUT1 expression | Promote glycolysis |
|  | IGF2BP3 | Recognizes m^6^A sites on c-MYC mRNA with LIN28B | Promote proliferation, migration, and glycolysis |
|  | IGF2BP1 | Binds m6A-modified c-MYC mRNA with LIN28B, enhancing stability | Promote development |
|  | METTL14 | Enhances stability of LHPP mRNA, which inhibits glycolysis via WNT pathway | Inhibit glycolysis and proliferation |
|  | FTO, YTHDF2 | FTO reduces YTHDF2 binding to m^6^A sites on PRKAA1 mRNA, increasing its stability | Inhibit glycolysis and promote apoptosis |
| **Lung Cancer** | METTL3 | LncRNA ABHD11-AS1 promotes glycolysis via ABHD11‐AS1/EZH2/KLF4 axis | Improve glycolysis in NSCLC |
|  | METTL3, YTHDF1 | DLGAP1-AS2 promotes c-MYC expression in an m^6^A-dependent manner | Improve glycolysis and proliferation |
|  | METTL3, ALKBH5, YTHDF1 | Act on ENO1 mRNA to regulate its expression | Improve glycolysis in LUAD |
|  | FTO, YTHDF1 | Decreased FTO increases m^6^A on c-MYC mRNA, YTHDF1 increases its expression | Improve glycolysis and proliferation in LUAD |
| **Breast Cancer** | WTAP | IL1β/TNFα (from neutrophils) act on ERK1/2-WTAP-ENO1 axis | Improve glycolysis |
|  | METTL3, YTHDF2 | METTL3/LATS1/YTHDF2 axis inhibits YAP/TAZ | Promote glycolysis and tumorigenesis |
|  | YTHDF1 | Hypoxia increases HIF-1α, which acts via miR-16-5p/YTHDF1/PKM2 axis | Improve genesis and metastasis |
|  | ALKBH5, YTHDF2 | ALKBH5 demethylates GLUT4 mRNA, YTHDF2 enhances its stability | Increase glycolysis, inducing resistance to HER2 targeted therapy |
| **Pancreatic Cancer** | YTHDF3 | Decreased stability of m^6^A-DICER1-AS1 reduces maturation of miR-5586-5p | Increase glycolytic gene expression, promoting proliferation and metastasis |
|  | IGF2BP3 | Recognizes m^6^A sites on c-MYC mRNA with LIN28B | Promote proliferation, migration, and glycolysis |
|  | IGF2BP1 | Binds m6A-modified c-MYC mRNA with LIN28B, enhancing stability | Promote development |
|  | METTL3, IGF2BP3 | m6A modification of Linc-UROD enhances its stability; Linc-UROD prevents ENO1/PKM degradation | Promote glycolysis and invasion |
|  | YTHDC1 | miR-30d (correlated with YTHDC1) targets RUNX1 to reduce GLUT1 and HK1 | Inhibit tumorigenesis and glycolysis |
|  | METTL3 | Promotes HK2 expression in an m^6^A-dependent manner | Promote glycolysis and perineural invasion (PNI) in PDAC |
| **Other Cancers** | Bladder: ALKBH5 | Low ALKBH5 increases m^6^A on CK2α mRNA, enhancing its stability | Reduce sensitivity to cisplatin chemotherapy |
|  | Glioblastoma (GBM): FTO | JPX binds PDK1 mRNA and enhances FTO-mediated demethylation | Induce resistance to temozolomide (TMZ) |
|  | Glioblastoma (GBM): ALKBH5 | Aerobic glycolysis induces exosomal circ_0072083, promoting ALKBH5-mediated NANOG expression | Induce resistance to temozolomide (TMZ) |
|  | General: FTO, ALKBH5 | GNRa-CSP12 disrupts Fe2+/Fe3+ balance, inactivating FTO/ALKBH5, reducing stability of GLUT3/PKM and immune checkpoint transcripts | Enhance efficacy of anti-PD-L1 immunotherapy |
|  | AML: FTO | R-2HG inhibits FTO, reducing stability of PFKP and LDHB transcripts | Inhibit glycolysis and proliferation |
|  | Melanoma: FTO | Dac51 inhibits FTO, reducing stability of JunB and C/EBPβ transcripts | Inhibit glycolysis and proliferation |
|  | Various: METTL3, IGF2BP3, YTHDC1 | IGF2BP3/YTHDC1 regulate NCAPH mRNA stability/nuclear export; NCAPH stabilizes β-catenin, promoting glycolysis and PD-L1 expression | Induce resistance to anti-PD-1 immunotherapy |
|  | METTL3, IGF2BP3 | m6A modification of Linc-UROD enhances its stability; Linc-UROD prevents ENO1/PKM degradation | Promote glycolysis and invasion |
|  | YTHDC1 | miR-30d (correlated with YTHDC1) targets RUNX1 to reduce GLUT1 and HK1 | Inhibit tumorigenesis and glycolysis |
|  | METTL3 | Promotes HK2 expression in an m^6^A-dependent manner | Promote glycolysis and perineural invasion (PNI) in PDAC |

**Table S2. Biopharmaceutical Companies and Clinical-Stage Programs Targeting the RNA Modification Landscape.**

| **Company** | **Location** | **Key Targets** | **Lead Product(s) & Approach** | **Indications** | **Highest Stage (as of 2025)** | **Clinical Trial Identifier / Reference** |
| --- | --- | --- | --- | --- | --- | --- |
| **Storm Therapeutics** | UK | METTL3, METTL1, ADAR1 | STC-15 (oral METTL3 inhibitor; related to STM2457) | Solid Tumors, AML | Phase I | NCT05584111 |
| **Accent Therapeutics** | USA | METTL3, ADAR1, DHX9 | ATX-559 (oral DHX9 inhibitor) | Solid Tumors, TNBC, AML | Phase I | NCT06625515 |
| **Gotham Therapeutics** | USA | METTL3, METTL14, ADAR1, QPCT/L | Undisclosed small-molecule inhibitors | Solid Tumors, Hematological Cancers | Preclinical / Phase I | NCT06395519 |
| **Epics Therapeutics** | Belgium, | METTL3 | EP102, EP282 (METTL3 inhibitors) | Solid Tumors, AML | Preclinical | [[76](#_ENREF_76)] |
| **H3 Biomedicine** | USA | SF3B Splicing Complex | H3B-8800 (splicing modulator) | AML, CML, MDS | Phase I | NCT02841540 |
|  |  |  |  |  |  |  |
| **Skyhawk Therapeutics** | USA | RNA Splicing Factors | SKY-0515 (small molecule splicing modulator) | Cancer, Huntington's Disease | Phase I | ACTRN12624000602527 |
| **Wave Life Sciences** | USA | RNA Editing (ADAR) | WVE-006 (AATD correction) | Alpha-1 Antitrypsin Deficiency | Phase I | NCT06405633 |
| **28/7 Therapeutics** | USA | Lin28/let-7 pathway, TUT4/TUT7 | Undisclosed Lin28 inhibitors | Solid Tumors | Discovery | <https://www.twentyeight-seven.com/> |
| **Ribometrix** | USA | RNA Structures (c-MYC, eIF4E) | Undisclosed small molecules | Solid Tumors | Discovery | [[77](#_ENREF_77)] |
| **Korro Bio** | USA | RNA Editing (ADAR) | OPERA™ platform for RNA editing | Liver & CNS Diseases | Discovery | <https://www.korrobio.com/> |
| **ProQR Therapeutics** | Netherland | AX-0810: for NTCP, AX-1412: for B4GALT1 | Axiomer™ ADAR RNA editing | Cholestatic liver (SLC10A1 gene), and CVD | Proposed clinical trial by 2025 | <https://www.proqr.com/> |

This table highlights the active drug discovery and development landscape targeting m^6^A regulators and other RNA-modifying enzymes, demonstrating the clinical translation of epitranscriptomic research. (Abbreviations: AML, Acute Myeloid Leukemia; DHX9, DExH-Box Helicase 9; TNBC, Triple-Negative Breast Cancer).

**Table S3. Pharmacological Modulators of the m^6^A Epitranscriptome.**

| **Target Class** | **Compound Name** | **Type** | **Key Target** | **Biological Effect** | **PMID** |
| --- | --- | --- | --- | --- | --- |
| **WRITER (METTL3) Inhibitors** |  |  |  |  |  |
|  | STM2457 (STC-15) | Competitive Inhibitor | METTL3 catalytic site (SAM-binding) | Anti-tumor; AML | 33902106, 37670178 |
|  | UZH1a | Competitive Inhibitor | Reduces global m^6^A ratio | Anti-leukemia; AML models | 34237194, 36692498 |
|  | CDIBA | Allosteric Inhibitor | METTL3 complex | Anti-proliferative; AML | 35040501 |
|  | Eltrombopag | Inhibitor | METTL3 complex | Anti-proliferative; AML | 35455436 |
|  | Quercetin | Natural Inhibitor | Inhibits tumor cell viability | Liver, Pancreatic Cancer | 35571106 |
| **WRITER (METTL3) Activators** |  |  |  |  |  |
|  | Piperidine/Piperazine-derivatives | Activator | METTL3-14-WTAP complex | Cell differentiation & proliferation; HEK293 cells | 30917327 |
| **ERASER Inhibitors** |  |  |  |  |  |
|  | FB23-2 | Competitive Inhibitor | FTO substrate-binding pocket | Anti-tumor; promotes differentiation/apoptosis; AML | 30991027 |
|  | R-2HG | Competitive Inhibitor | FTO; downregulates MYC/CEBPA | Anti-leukemia; AML | 29249359, 33434505 |
|  | CS1/CS2 | Inhibitor | Targets LILRB4, MYC; reprograms immune response | Attenuates leukemia self-renewal | 32531268 |
|  | Dac51 | Inhibitor | FTO; targets c-Jun, JunB | Enhances anti-tumor immunity; Melanoma | 33910046 |
|  | Rhein | Natural Inhibitor | FTO | Synergizes with other therapies; Leukemia | 23045983 |
|  | Meclofenamic Acid (MA2) | Inhibitor | FTO | Inhibits growth & self-renewal; GBM, NSCLC | 25452335, 35530301 |
|  | Entacapone | Inhibitor (Repurposed) | FTO-FOXO1 axis | Induces apoptosis; Esophageal Cancer | 30996080, 36534072 |
| **ERASER (ALKBH5) Inhibitors** |  |  |  |  |  |
|  | ALK-04 | Competitive Inhibitor | ALKBH5; reduces Mct4/Slc16a3 | Enhances anti-PD-1 efficacy; Melanoma, CRC | 32747553 |
|  | MV1035 | Inhibitor | ALKBH5; reduces CD73 | Anti-migratory/invasive; Glioblastoma | 31937477 |
|  | IOX1 | Broad-Spectrum Inhibitor | ALKBH5, other 2-OG oxygenases | Anti-tumor; Glioma | 36566230 |
| **READER Inhibitors** |  |  |  |  |  |
|  | **YTHDF2 Inhibitors** |  |  |  |  |
|  | DF-A7 | Inhibitor | YTHDF2 (IC~50~ = 53.83 nM) | Sensitizes CD8+ T-cells; improves anti-PD-1; Melanoma, NSCLC | 38820140 |
|  | DC-Y13 | Inhibitor | YTHDF2; targets SOCS-3 | Improves anti-PD-1 efficacy; Lung Metastasis | 37236197 |
|  | **IGF2BP1 Inhibitors** |  |  |  |  |
|  | Cucurbitacin B | Allosteric Inhibitor | IGF2BP1; destabilizes c-MYC | Anti-cancer; HCC | 36032766 |
|  | BTYNB | Inhibitor | IGF2BP1; destabilizes c-MYC | Anti-cancer; Ovarian Cancer, Melanoma | 28846937 |
|  | **IGF2BP2 Inhibitors** |  |  |  |  |
|  | CWI1-2 | Inhibitor | IGF2BP2; targets glutamine metabolism | Anti-leukemia; AML | 36306790 |
|  | JX5 | Inhibitor | IGF2BP2; targets NOTCH1 | Anti-leukemia; T-ALL | 35915142 |

* (Abbreviations: AML, Acute Myeloid Leukemia; CRC, Colorectal Cancer; HCC, Hepatocellular Carcinoma; NSCLC, Non-Small Cell Lung Cancer) *
